# Exploring dopamine as the master regulator of brain circuitry and mental health genome

**DOI:** 10.36922/gpd.6557

**Published:** 2025-06-03

**Authors:** Kenneth Blum, Eric R. Braverman, Alireza Sharafshah, Igor Elman, Kai-Uwe Lewandrowski, Abdalla Bowirrat, Albert Pinhasov, Panayotis K. Thanos, Mark S. Gold, Catherine A. Dennen, Edward J. Modestino, Rajendra D. Badgaiyan, David Baron, Brian Fuehrlein, Daniel Sipple, John Wesson Ashford, Keerthy Sunder, Milan Makale, Kevin Murphy, Nicole Jafari, Foojan Zeine, Aryeh R. Pollack, Alexander P.L. Lewandrowski, Jag Khalsa

**Affiliations:** 1 Division of Clinical Neurology, The Blum Institute of Neurogenetics and Behavior, Austin, Texas, United States of America; 2 Division of Personalized Pain Therapy Research and Education, Center for Advanced Spine Care of Southern Arizona, Tucson, Arizona, United States of America; 3 Department of Molecular Biology, Adelson School of Medicine, Ariel University, Ariel, Israel; 4 Division of Addiction Research and Education, Center for Sports, Exercise, and Mental Health, Western University of Health Sciences, Pomona, California, United States of America; 5 Cellular and Molecular Research Center, School of Medicine, Guilin University of Medical Sciences, Rasht, Iran; 6 Department of Psychiatry and Cambridge Health Alliance, Harvard Medical School, Cambridge, Massachusetts, United States of America; 7 Department of Orthopaedics, Fundación Universitaria Sanitas, Bogotá D.C., Colombia; 8 Department of Orthopaedics, Universidade Federal do Estado do Rio de Janeiro, Rio de Janeiro, Brazil; 9 Department of Psychiatry, Case Western Reserve University, School of Medicine, Cleveland, Ohio, United States of America; 10 Department of Pharmacology and Toxicology, Behavioral Neuropharmacology and Neuroimaging Laboratory on Addictions, Clinical Research Institute on Addictions, Jacobs School of Medicine and Biosciences, State University of New York at Buffalo, Buffalo, New York, United States of America; 11 Department of Psychiatry, Washington University School of Medicine, St. Louis, Missouri, United States of America; 12 Department of Family Medicine, Jefferson Health Northeast, Philadelphia, Pennsylvania, United States of America; 13 Department of Psychology, Curry College, Milton, Massachusetts, United States of America; 14 Department of Psychiatry, Yale University School of Medicine, New Haven, United States of America; 15 M Health Fairview University of Minnesota Medical Center, Minneapolis, Minnesota, United States of America; 16 Department of Psychiatry and Behavioral Sciences, Stanford University, Palo Alto, California, United States of America; 17 War Related Illness and Injury Study Center, VA Palo Alto Health Care System, Palo Alto, California, United States of America; 18 Department of Medicine, Riverside School of Medicine, University of California, Riverside, California, United States of America; 19 Department of Radiation Oncology, University of California San Diego, La Jolla, California, United States of America; 20 Department of Applied Clinical Psychology, The Chicago School of Professional Psychology, Los Angeles, United States of America; 21 Department of Health Science, California State University at Long Beach, Long Beach, California, United States of America; 22 Department of Medicine, School of Medicine, University of Maryland, Baltimore, Maryland, United States of America

**Keywords:** Dopamine, Addiction, Genetics, Brain reward cascade, Psychiatric disorders

## Abstract

Artificially increasing dopamine transmission is the common mechanism by which substances with addictive potential lead to addiction. A key area of research in neurobiology is the role of dopamine. Significant advancements have been made in uncovering the intracellular signaling pathways that mediate both dopamine’s immediate effects and its long-term influence on brain function. Recent discoveries have also highlighted specific molecules that could serve as potential therapeutic targets for neurological and psychiatric disorders. While understanding several important caveats, we believe dopamine acts as a master regulator of brain circuitry across major chromosomes mapping the mental health genome. This view may have important clinical relevance, emphasizing the critical role of dopaminergic activity across the genome. Importantly, we are cognizant that dopamine does not work in insolation, and its finite actions are due to a highly interactive network (known as the brain reward cascade), involving at least seven other major neurotransmitters.

## 1. Introduction

**Figure 1.**
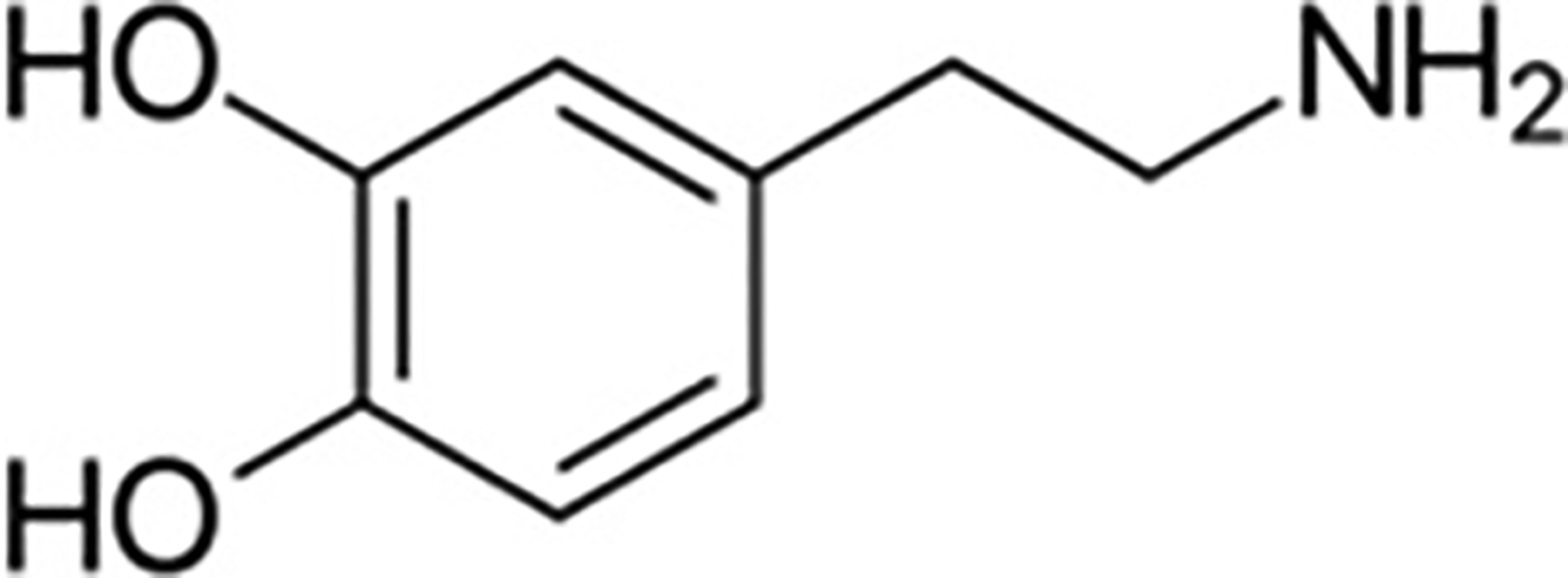
Chemical structure of dopamine

The organic chemical dopamine (3,4-dihydroxyphen ethylamine) belongs to the phenethylamine and catecholamine families and acts as a neuromodulatory molecule with several important roles in cellular functions. Approximately 80% of the catecholamine content in the brain is dopamine.^1^ It is an amine synthesized by decarboxylating its precursor, L-DOPA, produced in the brain and kidneys. Dopamine is also synthesized by most animals and plants.^2^ In the brain, dopamine is a neurotransmitter released by neurons, facilitating communication between nerve cells. The brain includes several distinct dopamine pathways, each playing unique roles in various neural functions. One unique role is the mesolimbic pathway, a critical pathway in rewarding motivated behavior.^3^ The anticipation of various rewards leads to an increase in dopamine levels in the brain.^4^ Several addictive substances either enhance release of dopamine or prevent its reuptake into neurons.^5^ Other dopamine pathways are important to motor control^5^ and regulation of secretion of hormones. These interconnected pathways and neuronal groups constitute the dopaminergic system, a neuromodulatory network that may function as a central regulator of brain circuitry, impacting a wide range of physiological and psychological processes. The impact extends across major brain regions and is integral to understanding the genetic underpinnings of mental health, thus suggesting its vital role in mapping mental health genomics (Figure 1).

## 2. Brain distribution and anatomical loci

Although the human brain contains only about 400,000 dopamine-producing neurons,^6^ with their cell bodies clustered in a few small regions, their axons extend widely throughout the brain, exerting a strong influence on their target areas.^7^

Annica Dahlström and Kjell Fuxe were the first who mapped dopaminergic cell groups in 1964, identifying ventral tegmental area (VTA), substantia nigra, posterior hypothalamus, zona incerta, arcuate nucleus, basal ganglia, and pars control. They also determined that motor skills are important functions of dopamine, which projects along the nigrostriatal pathway that runs to the dorsal striatum from the substantia nigra pars compacta 2.^8^

The main cluster of dopaminergic neurons in the VTA connects to the prefrontal cortex through the mesocortical pathway, while a smaller subset extends to the nucleus accumbens via the mesolimbic pathway. Together, these pathways form the mesocorticolimbic projection.^9^ Furthermore, the VTA sends dopaminergic projections to the amygdala, cingulate gyrus, hippocampus, and even the olfactory bulb.^9^ Mesocorticolimbic neurons are widely recognized for their central role in reward processing and motivation.^9^ In addition, dopamine is crucial for aversive learning, influencing multiple brain regions.^10–12^ Dopamine neurons from the posterior hypothalamus project to the spinal cord, impacting pain sensitivity.^13^ Dopamine neurons in the arcuate and periventricular nuclei of the hypothalamus form the tuberoinfundibular pathway that projects to the pituitary gland.^14^

The zona incerta, located between the arcuate and periventricular nuclei, extends projections to multiple hypothalamic regions and contributes to the regulation of gonadotropin-releasing hormone, a key factor in triggering the maturation of the male and female reproductive systems during puberty.^14^

Another area with dopamine-producing neurons is in the retina.^15^ These neurons, called amacrine cells, are axonless and release dopamine directly into the surrounding extracellular space.^15^ These cells are active during the day and become inactive at night. Retinal dopamine enhances cone cell function while inhibiting rod cells, improving color and contrast sensitivity in bright light but diminishing visual sensitivity in low-light environments.^15^

## 3. Genome and genes in the brain: A snapshot

Genes influence far more than traits like eye color or height; they play a crucial role in shaping every aspect of what it means to be human. They contain the instructions for creating the proteins that control all functions in our bodies. Some of these proteins are visible, like those forming our hair and skin, while others operate behind the scenes, regulating essential biological processes such as metabolism, immunity, and cell communication. Although nearly every cell in the body contains an identical set of genes, only a specific subset is active in each cell. When genes are active, they produce proteins through a process called gene expression. On the other hand, when genes are inactive, as seen with methylated histones, they remain silent or inaccessible, preventing protein production.

Approximately one-third of the 20,000 genes in the human genome are predominantly active in the brain, making it the area of the body with the highest gene expression. These genes are essential for the brain’s development and function, shaping how we move, think, feel, and behave. Alongside environmental factors, changes in these genes can also determine our susceptibility to certain diseases, particularly mental health disorders. For instance, the *ASPM* gene encodes a protein critical for the formation of new neurons in the developing brain. Mutations in this gene can result in microcephaly, a condition where the brain does not achieve its typical size.^16^ Another example is the *SOD1* gene, which produces a protein that helps protect neurons from DNA damage. This gene is thought to provide important insights into why neurons degenerate in the common “sporadic” form of amyotrophic lateral sclerosis, a type of disease with no known cause.^17^

The majority of single-gene mutations associated with rare neurological disorders, like Huntington’s disease, have been identified.^18^ However, much remains to be understood about the role of genetic variations in more common neuropsychiatric disorders like addiction. It is crucial to recognize that, for most individuals, the risk of developing complex polygenic disorders is influenced by a complex interaction between genes and environmental factors. In addition, although specific genetic variations, like single nucleotide polymorphisms (SNPs), have been associated with disease risk, the impact of any single variation is typically minimal. Changes in gene regulation, including those influenced by small RNAs and epigenetic factors, can also contribute significantly to the development of disease.

## 4. Dopamine across major chromosomes mapping

**Figure 2.**
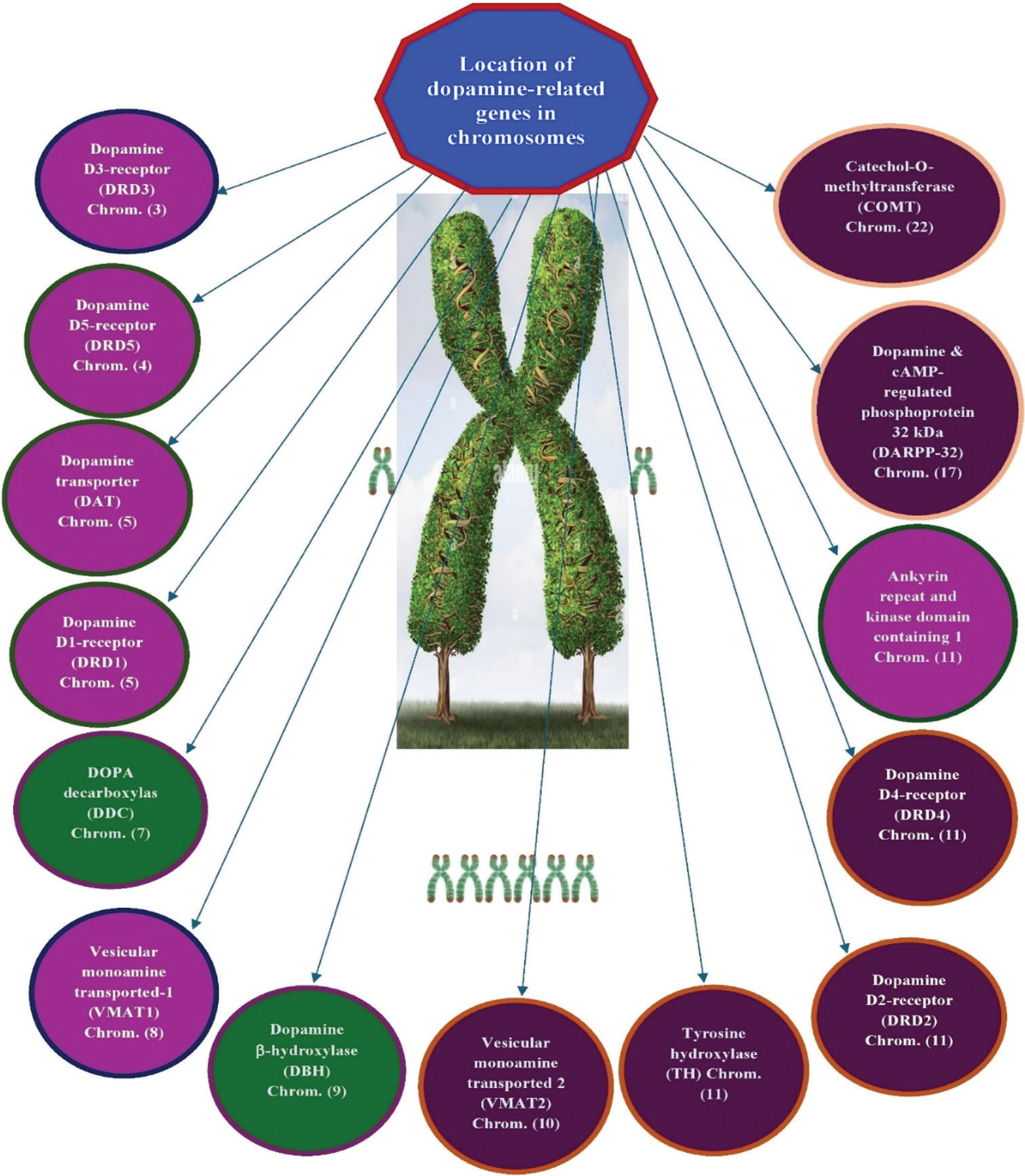
Location of dopamine-related genes on each chromosome as denoted from the human genome project

Figure 2 illustrates a schematic representation of the location of dopaminergic genes on each chromosome as denoted by the human genome project. This figure supports our concept that the dopamine molecule acts as a master regulator of brain circuitry functionality. The mapping of these genes across major chromosomes provides insights into the genetic underpinnings of mental health, highlighting dopamine’s central role in the genomics landscape of mental health.

Table 1 provides details of the specific chromosome, type of dopamine-linked gene, general chromosome function, and primary function of the dopamine gene.

## 5. Shared genetic underpinnings among psychiatric disordered: Links to dopamine-related genes

Midbrain dopaminergic neurons (MDNs) represent approximately 0.0005% of the brain’s neuronal population and are essential for mediating key functions such as cognition, food intake, and metabolism. MDNs are also believed to be involved in the pathophysiology of various neuropsychiatric disorders, which often present with multifactorial medical comorbidities, including metabolic diseases, leading to significantly increased morbidity and mortality.^32^ Psychiatric comorbidity, the coexistence of multiple psychiatric conditions,^33^ has gained significant attention due to its high prevalence and enduring effects.^2^ Individuals with co-occurring psychiatric conditions often experience poorer outcomes and significant impairments in multiple cognitive and behavioral areas.^34,35^ Many psychiatric disorders have their peak onset during adolescence, a period that often coincides with the emergence of comorbidity.^36,37^ For example, a population-based study on adolescent well-being found that 27.9% of participants aged 14 – 17 met the criteria for multiple diagnoses.^37^

The significant prevalence of comorbid mental disorders suggests potential neurobiological underpinnings shared across various psychopathologies. Increasing evidence supports the notion that many mental disorders may represent extreme manifestations along a continuous spectrum, with different conditions displaying overlapping deficits in multiple cognitive functions, as outlined by the research domain criteria framework.^38^

There is growing recognition that various psychiatric disorders stem from common neural pathways, affecting overlapping brain systems.^39^ Park *et al*.^39^ conducted a study examining the multiscale neural contextualization of cortical morphology alterations across six major psychiatric disorders: autism spectrum disorder, attention-deficit/hyperactivity disorder, major depressive disorder, obsessive-compulsive disorder, bipolar disorder, and schizophrenia. By aggregating disease-related effects on magnetic resonance imaging (MRI)-derived cortical thickness from six ENIGMA working groups, encompassing 28,546 participants (12,876 patients and 15,670 controls), they identified a cortex-wide dimension of morphological changes. This dimension followed a sensory-fugal pattern, with paralimbic regions exhibiting the most consistent alterations across disorders. In addition, the shared disease dimension was strongly associated with cortical microstructure gradients and variations in serotonin and dopamine neurotransmitter systems.

Recent large-scale imaging studies suggest that psychiatric disorders have overlapping biological substrates. Tu *et al*.^32^ conducted an analysis of a resting-state functional MRI dataset comprising 100 patients each with schizophrenia, bipolar I disorder, bipolar II disorder, and major depressive disorder, along with 100 healthy controls. Their findings revealed shared connectomic abnormalities across cortical and subcortical structures in the four patient groups. Affected regions included the bilateral thalamus, cerebellum, frontal pole, supramarginal gyrus, postcentral gyrus, lingual gyrus, lateral occipital cortex, and parahippocampus. Further analysis of pairwise functional connectivity among these regions indicated that the psychiatric disorders exhibited similar patterns of connectivity disruptions, characterized by sensory/subcortical hyperconnectivity, association/subcortical hypoconnectivity, and sensory/association hyperconnectivity.

Genome-wide association studies (GWAS) conducted by the Psychiatric Genomics Consortium have identified common SNPs across five major psychiatric disorders: schizophrenia, bipolar disorder, autism spectrum disorder, major depressive disorder, and attention-deficit/hyperactivity disorder.^40^ In addition, analyses of resting-state functional connectivity MRI and whole-brain connectomics in schizophrenia, bipolar I disorder, bipolar II disorder, and major depressive disorder, have revealed shared patterns of neural abnormalities among patient groups, differentiating them from healthy controls.^41^ These findings pose a challenge to the current diagnostic classification system and suggest a shift in psychiatry from a focus on descriptive syndromes to a nosology informed by the underlying causes of disease.

Dysregulated signaling within mesocorticolimbic dopamine-related circuits is increasingly recognized as a key neuropathological mechanism underlying various psychiatric disorders. Research indicates that alterations in these circuits are closely associated with the pathophysiology of multiple conditions. For instance, a study by Nakamura *et al*.^42^ revealed that excitatory shell-to-core connectivity was greater in all patient groups compared to controls. The study further revealed that inhibitory shell-to-VTA and shell-to-mPFC connectivity was stronger in individuals with autism spectrum disorder compared to controls. Moreover, while VTA-to-core and VTA-to-shell connections were excitatory in the ASD group, they were inhibitory in individuals with major depressive disorder and schizophrenia. Structural connectivity deficits in ADHD may also stem from a common etiological mechanism, involving disruptions in synaptic potentiation and pruning modulated by dopamine and other factors during development. Collectively, these findings suggest that core ADHD symptoms may arise from dysregulated cortical plasticity in early brain development, resulting in altered corticocortical connectivity patterns that can persist into adulthood.^43^

In addition, research has demonstrated that multivariate connectivity in the sensorimotor putamen is altered in individuals with binge eating disorder and bulimia nervosa, with the extent of the alteration correlating with the severity of disordered eating behaviors. These connectivity abnormalities were associated with changes in mean diffusivity in the sensorimotor putamen as well as reduced basal dopamine D2/3 receptor binding potential in the striatum. These findings are consistent with prior studies reporting microstructural changes and disruptions in dopamine signaling, which are known to influence habit learning in animal models.^44^

Similarly, Xue *et al*.^45^ investigated the nucleus accumbens of individuals with opioid use disorder (OUD) and found that rhythmic transcripts peaked either in the evening or near sunrise, aligning with neurotransmission systems involving opioids, dopamine, and GABA. Co-expression network analysis further reinforced these findings, identifying OUD-specific modules enriched for dopaminergic, GABAergic, and glutamatergic synaptic functions. In addition, rhythmic transcript changes in both the dorsolateral prefrontal cortex and nucleus accumbens of OUD subjects were associated with genomic loci linked to sleep-related traits, such as sleep duration and insomnia. These results underscore a connection between transcriptional rhythm disruptions in key neurotransmitter systems and sleep-related traits in opioid addiction.

Building on the discovery linking dopamine D2 receptor gene polymorphism to severe alcoholism, there has been a surge of research in psychiatric, behavioral addiction, and neurogenetics literature.^46^ Reviews highlight the essential role of dopaminergic pathways and resting-state functional connectivity within brain reward circuits. A key concept proposed is reward deficiency syndrome, a condition where disruptions in the brain’s reward cascade – either genetically or environmentally induced through epigenetic changes – contribute to both substance and non-substance addictive behaviors.

A deeper understanding of shared common mechanisms will ultimately improve diagnosis, treatment, and relapse prevention. Although the complexities of behavioral addiction remain only partially understood, we are starting to ask the right questions. Through extensive global research, we are progressing toward developing strategies to restore balance in the brain’s reward systems, providing hope for a future where individuals can overcome addiction and regain a life of fulfillment and well-being.^47^

Heritable behaviors and their consistent execution are believed to be governed by genetically encoded programs.^48^ Fiore *et al*.^48^ investigated the functional anatomy of the insect central complex and the vertebrate basal ganglia, revealing their shared role in selecting and maintaining adaptive behaviors. Comparative analyses showed that these circuitries share similar lineage relationships within clusters of functionally integrated neurons. Both systems are modulated by dopamine signaling, which also affects memory-like processes. The notable similarities between the central complex and basal ganglia suggest the presence of evolutionarily conserved computational mechanisms that underlie action selection.

Qian *et al*.^49^ investigated the impact of DRD4 regulation on resting-state brain activity in children with ADHD using regional homogeneity and functional connectivity analyses. Resting-state functional MRI data from 49 children with ADHD showed that those carrying the *DRD4 2R* allele exhibited reduced regional homogeneity bilaterally in the posterior cerebellar lobes but increased regional homogeneity in the left angular gyrus. Compared to individuals with the *DRD4 4R/4R* genotype, those with the *2R* allele displayed decreased functional connectivity between the left angular gyrus and the left striatum, right inferior frontal gyrus, and bilateral cerebellar lobes. Conversely, increased FC was found in the left superior frontal gyrus, medial frontal gyrus, and rectus gyrus.

These findings establish a link between *DRD4* polymorphisms and distinct patterns of brain activity, particularly within the frontal-striatal-cerebellar circuitry. In addition, the catechol-*O*-methyltransferase enzyme, which plays a key role in regulating dopamine flux, contains a well-documented functional polymorphism – val(158) met. This polymorphism has been shown to influence prefrontal function and working memory capacity, and it has also been implicated in anxiety and emotional dysregulation.^50,51^

## 6. Summary

The extensive overlap among major psychiatric disorders is notable, encompassing genetic variants, brain structure, function, and clinical symptoms. Bourque *et al*.^52^ have systematically reviewed this phenomenon by comparing the degree of similarity between psychiatric conditions across available data sources. By searching PubMed and EMBASE from January 2009 to September 2022, Bourque *et al*.^52^ analyzed 28 eligible studies that explored similarities among schizophrenia, bipolar disorder, major depressive disorder, autism spectrum disorder, and ADHD. Their review encompassed 2975 studies that employed similarity measures based on SNPs, gene-based analyses, gene expression, as well as structural and functional neuroimaging data. Notably, the majority of correlations (88.6%) across disorders were positive. By estimating genetic correlation levels, they found that the likelihood of another psychiatric diagnosis in first-degree relatives was consistently lower than the rates observed in population studies. These findings highlight the significant, though not exclusive, role of genetic and neurobiological factors in the diagnostic overlaps commonly encountered in clinical practice.

Furthermore, the high comorbidity rates among psychiatric disorders observed in epidemiological studies^53–57^ mirror the positive, non-zero genetic correlations identified in large-scale genetic analyses.^58–61^ To identify shared biological processes underpinning this observed phenotypic and genetic covariance and enhance molecular characterization of general psychiatric disorder liability, Romero *et al*.^62^ employed multiple strategies to uncover pleiotropic – cross-trait-associated – SNPs, genes, and biological pathways. While it seems plausible that there are shared common mechanisms across psychiatric disorders, Romero *et al*.^62^ concluded that the identification of these shared biological mechanisms remains challenging due to differences in power and genetic architecture among the various psychiatric conditions.^63–66^

Genome sequencing has identified more than 300 million genetic variations in human populations, with over 90% consisting of SNPs. The remaining variations include short deletions, insertions, and small structural variants. Through GWAS, hundreds of thousands of these variants have been linked to specific traits and diseases. Interestingly, only about 5% of disease-associated SNPs are found within gene-coding regions, where they may impact gene expression or protein function. The remaining 95% are located in non-coding regions, which account for 98% of the genome. Initially, the role of these non-coding SNPs – many of which are distant from any genes – was unclear. However, it was later discovered that gene promoters often interact with distant regulatory elements, influencing gene expression.^67^

Importantly, disease-associated SNPs are enriched at the millions of gene regulatory elements within the non-coding sequences of the genome, suggesting they function as gene-regulating variants. Identifying genetic signatures that contribute to a specific disease is the first step in designing rational therapeutic interventions. With the understanding of this and many other unknown complications, our perspective raises the interesting question as to whether the actual dopaminergic function of the brain has the heuristic value as the master commander of brain circuitry functionality, a topic that should be investigated by mapping major chromosomes to depict the genomic landscape of mental health.

The brain houses two main groups of dopamine neurons. One group, located in the arcuate nucleus of the hypothalamic median eminence, is involved in neuroendocrine regulation. The other group, found in the ventral mesencephalon, projects to the forebrain. Although dopamine neurons account for fewer than 1 in 100,000 brain neurons, they are crucial in regulating essential brain functions. Proper dopamine function is vital for the normal operation of regions they innervate, including those involved in motor behavior, motivation, working memory, and pleasure-seeking behaviors. Dopamine neurons are also central to the brain’s reward system, influencing the learning of various behaviors. The loss of nigrostriatal dopamine neurons leads to Parkinson’s disease, while dopamine receptor blockade has therapeutic effects in treating psychosis.^68^

It is noteworthy that futuristic thinking in terms of patient care should firmly consider the role of circadian rhythm, for not only its dopaminergic function but also the genetic and epigenetic interface concerning mood disorders. In fact, mood disorders have been linked with alterations in the expression of circadian rhythm genes such as *Clock*, *Bmal1*, and *Per*. It is well-known that the half-life, metabolism, absorption, biodistribution, and effects on target organs are all affected by the body’s circadian rhythms.^69^ Specifically, Blum’s group suggested that circadian rhythm dysfunction, neuroadaptation in the reward circuits, and changes, especially in clock gene expression in the mesolimbic areas, undoubtedly affect substance use disorder, and such chronotherapy has realistic benefits in the treatment approaches for all reward deficiencies and unwanted behaviors.^70^ Along these lines, in terms of clinical utility, it is important to pay more attention to somatic issues, for example, a combination of brain and intestinal disorders (colitis, irritable bowel disorder, etc.), very common in autism spectrum disorder. Furthermore, gastrointestinal disturbances can contribute to psychological stress. It is also well-established that the gut microbiota synthesizes neuroactive compounds, including DOPA, which can cross the blood–brain barrier and subsequently convert to dopamine within the central nervous system. This important topic is sometimes ignored by the neuroscience community focused on dopaminergic and other neurotransmitter interactions and mechanisms; microbiota-gut-brain axis is considered in detail in a number of recent publications.^71,72^

## 7. Conclusion

The artificial increase in dopamine transmission is a central mechanism by which drugs of abuse induce addiction. Understanding the mechanisms of dopamine action remains one of the primary goals of neurobiology. Significant progress has been made in identifying the intracellular signaling pathways that mediate both the immediate effects of dopamine and its long-term impact on brain function. Recent research has led to the identification of key molecules that could serve as potential targets for future treatments in psychiatry and neurology. Despite several important caveats, we believe that our perspective may have important translational clinical relevance to help the scientific community to consider the unique and major role of dopaminergic activity across the entire genome. Importantly, we are cognizant that dopamine does not work in insolation and its finite actions are due to a highly interactive network (known as the brain reward cascade), involving at least seven other major neurotransmitters.

## Figures and Tables

**Table 1.** Dopamine genes and chromosomal function map

Chromosome	Dopamine gene	Chromosome properties	Gene function	References
3	*DRD3*	Spans 201 million bp; comprises about 6.5% of total cellular DNA.	This gene codes for the D3 subtype of the dopamine receptor, which reduces adenylyl cyclase through inhibitory G-proteins. DRD3 is found in brain regions that are evolutionarily older, where it helps regulate cognitive and emotional functions.	19
4	*DRD5*	Spans more than 193 million bp; comprises 6% to 6.5% of total cellular DNA.	This gene encodes the D5 subtype of the dopamine receptor, which has a tenfold higher affinity for dopamine compared to the D1 subtype.^6^ It promotes the synthesis of cAMP through the activation of the Gαs/olf family of G proteins.^7,8^	20
5	*DRD1*	Spans about 182 million bp; comprises almost 6% of total cellular DNA.	The D1 receptor is positively coupled to cAMP production, playing a role in memory, learning, neuron growth, reward system, and locomotor activity. It also modulates events mediated by the dopamine receptor D2.	21
5	*DAT1*	Spans about 182 million bp; comprises almost 6% of total cellular DNA.	DAT is a membrane protein responsible for removing dopamine from the synaptic cleft, thereby regulating its signaling and influencing cognition and reward.	22
7	*DDC*	Spans more than 172 million bp; comprises 5.5% to 6% of total cellular DNA. It comprises the major histocompatibility complex, of which over 100 genes are related to the immune response and organ transplantation.	Aromatic L-amino acid decarboxylase (AADC or AAAD), also referred to as DDC, tryptophan decarboxylase, and 5-hydroxytryptophan decarboxylase, is a rate-limiting enzyme in treatments involving L-DOPA or SSRIs.	23
8	*VMAT1*	Spans about 146 million bp; comprises between 4.5% and 5.0% of total cellular DNA. About 8% of its genes are linked to brain development and function; about 16% are involved in cancer. A 15-megabase region exhibit a high mutation rate, showing significant divergence between human and chimpanzee suggesting its contribution to the evolution of the human brain.	VMAT1, also known as CGAT or SLC18A1, is encoded by the *SLC18A1* gene. It is a membrane protein responsible for transferring monoamines, such as norepinephrine, epinephrine, dopamine, and serotonin, between the cytosol and synaptic vesicles.	24
9	*DBH*	Spans about 150 million bp; comprises 4.0% to 4.5% of total cellular DNA.	DBH, also known as dopamine beta-monooxygenase, is an enzyme (EC 1.14.17.1) encoded by the *DBH* gene in humans. It catalyzes the conversion of dopamine into norepinephrine.	25
10	*VMAT2*	Spans about 134 million bp; comprises 4% to 4.5% of total cellular DNA.	SLC18A2, also known as VMAT2, is a protein encoded by the *SLC18A2* gene in humans. It is an integral membrane protein responsible for transporting monoamines (dopamine, norepinephrine, serotonin, and histamine) from the cellular cytosol into synaptic vesicles.	26
11	*TH*	Spans about 135 million bp; comprises 4% to 4.5% of total cellular DNA; one of the most gene-rich, and disease-rich, chromosomes in the human genome.	TH, or tyrosine 3-monooxygenase, is the rate-limiting enzyme in the synthesis of catecholamines. It catalyzes the conversion of L-tyrosine to L-3,4-dihydroxyphenylalanine (L-DOPA) using molecular oxygen (O_2_), iron (Fe^2+^), and tetrahydrobiopterin as cofactors.^5,6^ L-DOPA serves as a precursor to dopamine, which, in turn, is a precursor for the crucial neurotransmitters such as norepinephrine (noradrenaline) and epinephrine (adrenaline). TH catalyzes the rate-limiting step in the synthesis of these catecholamines.	27
11	*DRD2*	Spans about 135 million bp; comprises 4% to 4.5% of total cellular DNA; one of the most gene-rich, and disease-rich, chromosomes in the human genome.	Dopamine receptor D2, also known as D2R, is a protein encoded by the *DRD2* gene in humans. The polymorphism Taq 1A (rs1800497) was once thought to be associated with the *DRD2* gene, but it actually resides in exon 8 of the *ANKK1* gene. Genome-wide association studies related to depression, suicidal ideation, and substance use disorder consistently identify the *DRD2* gene as a top candidate.	28
11	*DRD4*	Spans about 135 million bp; comprises 4% to 4.5% of total cellular DNA; one of the most gene-rich, and disease-rich, chromosomes in the human genome.	The dopamine receptor D4 is a dopamine D2-like G protein-coupled receptor encoded by the *DRD4* gene located on chromosome 11 at 11p15.5. Like other dopamine receptor subtypes, the D4 receptor is activated by the neurotransmitter dopamine. It has been implicated in various neurological and psychiatric conditions, including schizophrenia, bipolar disorder, ADHD, addictive behaviors, Parkinson’s disease, and eating disorders like anorexia nervosa. A weak association has also been suggested between the *DRD4* gene and borderline personality disorder.	29		
17	*DARPP-32*	Spans more than 84 million bp; comprises 2.5% to 3% of total cellular DNA.	PPP1R1B, also known as dopamine- and cAMP-regulated neuronal phosphoprotein (DARPP-32), is a protein encoded by the *PPP1R1B* gene in humans. DARPP-32 plays a crucial role in dopamine-dependent striatal synaptic plasticity, potentially acting as a dopamine-dependent gating mechanism for calcium/CaMKII signaling.	30		
22	*COMT*	The second smallest human chromosome, spanning about 51 million DNA bp; comprises 1.5 – 2% of total cellular DNA.	COMT is an enzyme that degrades catecholamines (dopamine, epinephrine, and norepinephrine) and other substances with a catechol structure. In humans, the homozygous Val variant metabolizes dopamine at up to 4 times the rate of its methionine counterpart. The Val158Met polymorphism is believed to influence cognition by modulating dopamine signaling in the frontal lobes.	31		

Abbreviations: ADHD: Attention-deficit/hyperactivity disorder; bp: Base pairs; CGAT: Chromaffin granule amine transporter; COMT: Catechol-*O*-methyltransferase; DAT: Dopamine transporter; DBH: Dopamine beta-hydroxylase; DDC: DOPA decarboxylase; DRD: Dopamine receptor D; PPP1R1B: Protein phosphatase 1 regulatory subunit 1B; SLC18A1: Solute carrier family 18 member 1; TH: Tyrosine hydroxylase; VMAT1: Vesicular monoamine transporter 1.
